# New evidence-based A1, A2, A3 alarm time zones for transferring thrombolysed patients to hyper-acute stroke units: faster is better

**DOI:** 10.1007/s10072-019-03901-8

**Published:** 2019-04-27

**Authors:** Thang S. Han, Giosue Gulli, Brendan Affley, David Fluck, Christopher H. Fry, Christopher Barrett, Puneet Kakar, Sapna Sharma, Pankaj Sharma

**Affiliations:** 1 Institute of Cardiovascular Research, Royal Holloway, University of London, TW20 0EX Egham, UK; 2 Department of Stroke, Ashford and St Peter’s NHS Foundation Trust, KT16 0PZ Chertsey, UK; 3 Department of Cardiology, Ashford and St Peter’s NHS Foundation Trust, KT16 0PZ Chertsey, UK; 4 School of Physiology, Pharmacology and Neuroscience, University of Bristol, BS8 1QU Bristol, UK; 5 Department of Stroke, NHS Frimley Health Foundation Trust, GU16 7UJ Frimley, UK; 6 Department of Stroke, Epsom and St Helier University Hospitals NHS Trust, SM5 1AA Surrey, UK; 7 Department of Clinical Neuroscience, Imperial College Healthcare NHS Trust, W12 0HS London, UK

**Keywords:** Evidence-based medicine, ROC analysis, Ischaemic stroke, Thrombolysis, tPA, Stroke units

## Abstract

**Objectives:**

The National Institute of Health and Clinical Excellence and The Royal College of Physicians recommend transferring thrombolysed patients with stroke to a hyperacute stroke unit (HASU) within 4 h from hospital arrival (T_Arrival-HASU_), but there is paucity of evidence to support this cut-off. We assessed if a shorter interval within this target threshold conferred a significant improvement in patient mortality.

**Design:**

We conducted a retrospective analysis of prospectively collected data from the Sentinel Stroke National Audit Programme.

**Setting:**

Four major UK hyperacute stroke centres between 2014 and 2016.

**Participants:**

A total of 183 men (median age = 75 years, IQR = 66–83) and 169 women (median age = 81 years, IQR = 72.5–88) admitted with acute ischaemic stroke.

**Main outcome measures:**

We evaluated T_Arrival-HASU_ in relation to inpatient mortality, adjusted for age, sex, co-morbidities, stroke severity, time between procedures, time and day on arrival.

**Results:**

There were 51 (14.5%) inpatient deaths. On ROC analysis, the AUC (area under the curve) was 61.1% (52.9–69.4%, *p* = 0.01) and the cut-off of T_Arrival-HASU_ where sensitivity equalled specificity was 2 h/15 min (intermediate range = 30 min to 3 h/15 min) for predicting mortality. On logistic regression, compared with the fastest T_Arrival-HASU_ group within 2 h/15 min, the slowest T_Arrival-HASU_ group beyond upper limit of intermediate range (≥ 3 h/15 min) had an increased risk of mortality: 5.6% *vs.* 19.6%, adjusted OR = 5.6 (95%CI:1.5–20.6, *p* = 0.010).

**Conclusions:**

We propose three new alarm time zones (A1, A2 and A3) to improve stroke survival: “A1 Zone” (T_Arrival-HASU_ < 2 h/15 min) indicates that a desirable target, “A2 Zone” (T_Arrival-HASU_ = 2 h/15 min to 3 h/15 min), indicates increasing risk and should not delay any further, and “A3 Zone” (T_Arrival-HASU_ ≥ 3 h/15 min) indicates high risk and should be avoided.

## Introduction

Thrombolysis is an effective treatment for acute ischaemic stroke [1] and is widely performed in hyperacute stroke units (HASUs) [2, 3]. Door to needle time has been a major focus of research [4, 5], but the time taken to transfer patients from hospital arrival to HASU is less studied and indeed may be as important. The National Institute of Health and Clinical Excellence (NICE) [6] and Royal College of Physicians (RCP) recommend patients presenting with an acute stroke to be transferred to a HASU within 4 h from hospital arrival (T_Arrival-HASU_) [7]. This time window covers the time taken from the point at which the patient arrives hospital to brain imaging, then to intravenous thrombolysis, and followed by the transfer to HASU; thus, T_Arrival-HASU_ partly depends on the speed at which the patient receives brain imaging and thrombolysis.

The T_Arrival-HASU_ span of 4 h is relatively long but is related to the time needed to administer recombinant tissue plasminogen activator (rtPA). Some centres provide thrombolysis within the CT scanning unit to minimise delay while others may return the patient to the Emergency department or send to HASU for such an intervention [7].

In this study, we attempted to determine whether a more detailed subset of T_Arrival-HASU_ could be derived within the recommended target threshold of 4 h among patients undergoing thrombolysis for acute ischaemic stroke. This should provide an evidence-based target time for T_Arrival-HASU_ in the management of these patients.

## Methods

### Study design, participants and setting

We conducted a retrospective analysis of prospectively collected data from the Sentinel Stroke National Audit Programme (SSNAP) which is the national register of stroke care, comprising clinical characteristics and care quality of patients admitted to acute care hospitals in England and Wales [8]. The data were collected prospectively from the time of admission up to 6 months after stroke in patients admitted to four major UK hyperacute stroke centres in Surrey County between January 2014 and February 2016 [9, 10]. In order to test our objectives against the 4-h transfer target from the point of arrival to HASU set out by NICE [6] and RCP [7] for thrombolysed patients, the present study selected only those who were thrombolysed.

HASUs are located in each of the four study centres, providing HASU management but not endovascular services. Patients eligible for endovascular thrombolysis started intravenous thrombolysis locally and were then transferred to the regional tertiary centre, after discussion with the interventional neuroradiologists, for endovascular treatment. The SSNAP database does not collect information on patients receiving endovascular treatment in the tertiary centre. Audits at the time of data collection showed < 10% of patients were referred for endovascular treatment. Thus, patients with large artery occlusion, and not eligible for endovascular treatment, were included in this study. Because patients who had endovascular treatment were not included in this study, results refer only to patients who had intravenous treatment.

SSNAP has approval from the Confidentiality Advisory Group of the Health Research Authority to collect patient data under section 251 of the National Health Service Act 2006. No additional ethical approval was sought [9, 10].

### Socio-demographic factors and medical history

Demographic data were collected and documented by stroke consultants and stroke nurse specialists including age at arrival, gender, past medical history (atrial fibrillation, hypertension, congestive heart failure, diabetes mellitus, previous stroke and drug history) and time points from onset of symptoms to hospital arrival, brain imaging, thrombolysis and transfer times to HASU. In addition, details of new cases of urinary tract infection and pneumonia acquired in hospital within 7 days of admission were documented.

### Stroke diagnosis and severity

Stroke was diagnosed based on clinical presentation and brain imaging. The severity of stroke symptoms at arrival and 24 h post-thrombolysis was assessed by the National Institutes of Health for Stroke Scale (NIHSS) with a score range from no symptoms to severe stroke symptoms (NIHSS score = 0 to 42).

### Disability and mortality

Degree of disability or dependence with daily activities was assessed by a modified Rankin Scale (mRS), ranging from no symptoms to severe symptoms (mRS score = 0 to 5) and mortality (mRS score = 6).

### Thrombolysis

Thrombolysis using the fibrinolytic agent alteplase (rtPA) was considered for patients who fulfilled criteria for treatment including time from onset, confirmed diagnosis of ischaemic stroke and without contra-indications.

### Categorisation of variables

Dichotomisation was applied for hypertension, congestive heart failure, atrial fibrillation, diabetes and inpatient mortality according to the presence or absence of history of the condition. Moderately severe to severe stroke on arrival or 24 h post-thrombolysis was defined as a NIHSS score ≥ 16 and moderately severe to severe disability on discharge for those with mRS score = 4 and 5.

### Statistical analysis

We initially performed receiver operating characteristic (ROC) curve analysis to determine (1) the association of T_Arrival-HASU_ with mortality as indicated by area under the curve (AUC) and (2) the cut-offs of T_Arrival-HASU_ (d_0_) where sensitivity equals specificity for identifying mortality using the two-graph ROC plot technique [11, 12]; d_0_ was identified by interpolating from the intersection where sensitivity equals specificity (θ_0_) and limits of intermediate range (IR) from the point where sensitivity (lower limit) and specificity (upper limit) equal 95%. We then conducted multivariable logistic regression analysis to estimate the risk of inpatient mortality (dependent variable) from slower T_Arrival-HASU_ (above the d_0_ and above upper limit of IR) compared with the referent group of hyper-fast T_Arrival-HASU_ within d_0_ (predictive variables). The results were presented in four models; *model 1*: unadjusted, *model 2*: adjusted for age, sex and co-morbidities, *model 3*: additional adjustment to model 2 for stroke severity on arrival, time from symptom onset to hospital arrival (door time), from arrival to brain imaging and from arrival to thrombolysis (door to needle), and *model 4*: additional adjustment to model 3 for time of day and day of week on arrival. Results are expressed in odds ratios (OR) and 95% confidence intervals (CI). Most variables had no missing data, which were handled in analysis using a ‘listwise deletion of missing data’ approach [13]. Analyses were performed using SPSS V.23.0 (SPSS Inc., Chicago, Illinois, USA). The null hypothesis was rejected when *p* < 0.05.

## Results

**Fig. 1:**
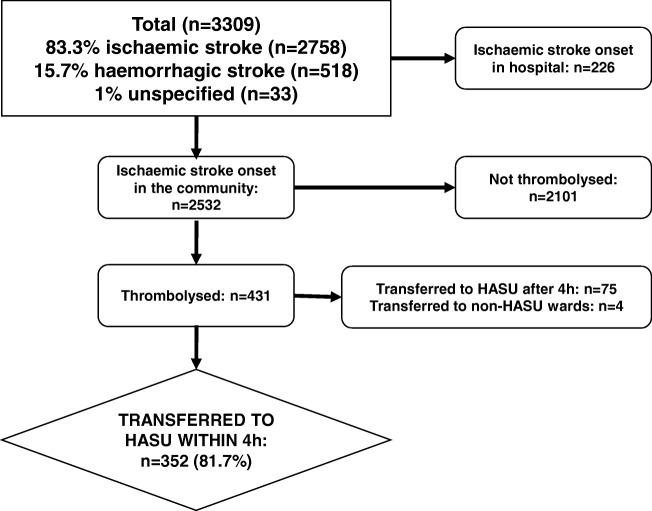
Flowchart of patient cohort

Of the 3309 patients collected in the database, 2758 (83.3%) patients presented with ischaemic stroke; of the remainder 518 (15.7%) patients had a haemorrhagic stroke and 33 (1.0%) were unspecified. Among patients with ischaemic stroke, 431 (15.6%) underwent thrombolysis, in whom 352 (183 men and 169 women) achieved the T_Arrival-HASU_ target within 4 h; these patients were selected for analysis in relation to inpatient mortality (Fig. 1).

Median and interquartile range (IQR) age was 75 (66–83) years in men and 81 (72.5–88) years in women. The median time from symptom onset to hospital arrival was 1 h/9 min (50 min to 1 h/39 min), arrival to brain imaging was 19 min (13–28 min), arrival to thrombolysis was 50 min (34 min to 1 h/8 min) and T_Arrival-HASU_ was 3 h/00 min (2 h/22 min to 3 h/36 min). In total, there were 51 (14.5%) inpatient deaths (Table 1).

**Table 1:** Characteristics of 352 patients admitted with ischaemic stroke who received thrombolysis and transferred to HASU within 4 h on arrival

	Median	Interquartile range
Age of men (years)	75.0	66.0–83.0
Age of women (years)	81.0	72.5–88.0
Time between symptom onset and arrival (h/min)	1:09	0:50–1:39
Time between arrival to brain imaging (h/min)	0:19	0:13–0:28
Time between arrival to thrombolysis (h/min)	0:50	0:34–1:08
Time between arrival and transfer to HASU (h/min)	3:00	2:22–336
	n	Proportion (%)
Men/women	183:169	52:48
First stroke/recurrent stroke	279:73	79.3:20.7
Atrial fibrillation	70	19.9
Hypertension	192	54.5
Congestive heart failure	12	3.4
Diabetes	55	15.6
Inpatient mortality	51	14.5

**Fig. 2:**
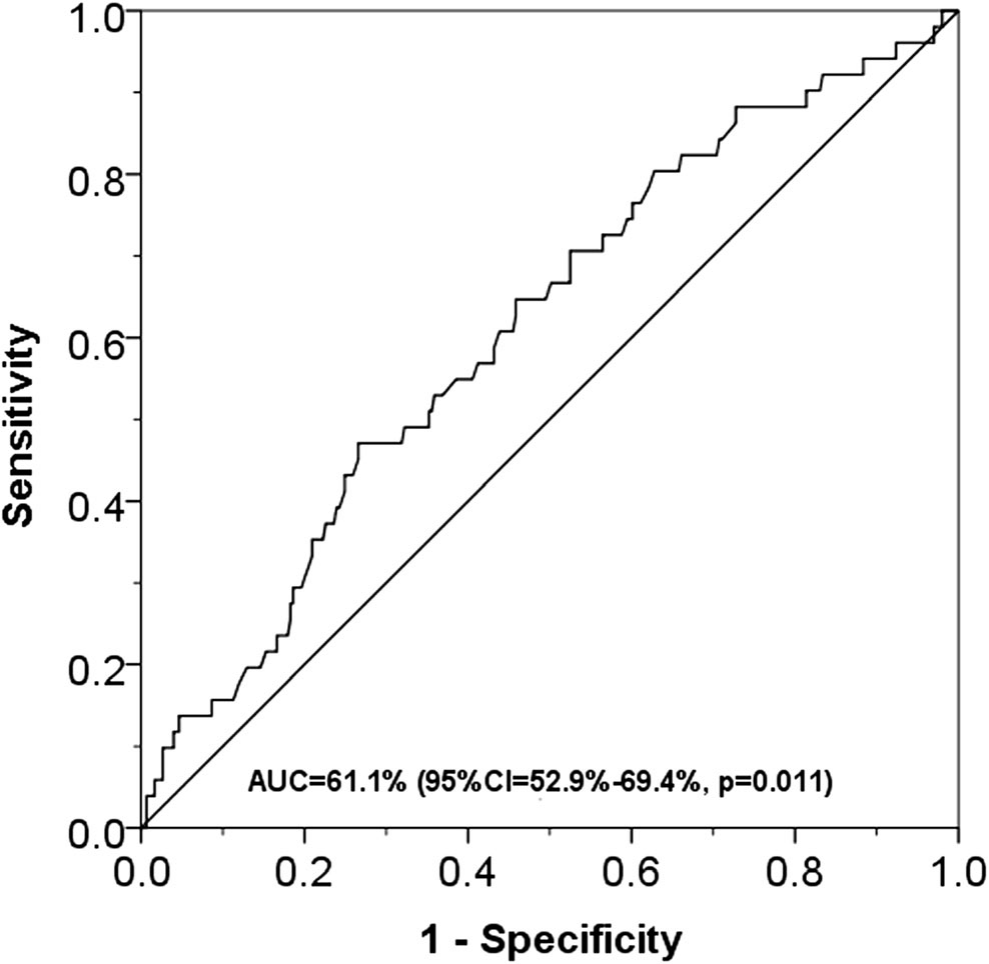
ROC curve for identifying inpatient mortality from different transfer time from hospital arrival to HASU

**Fig. 3:**
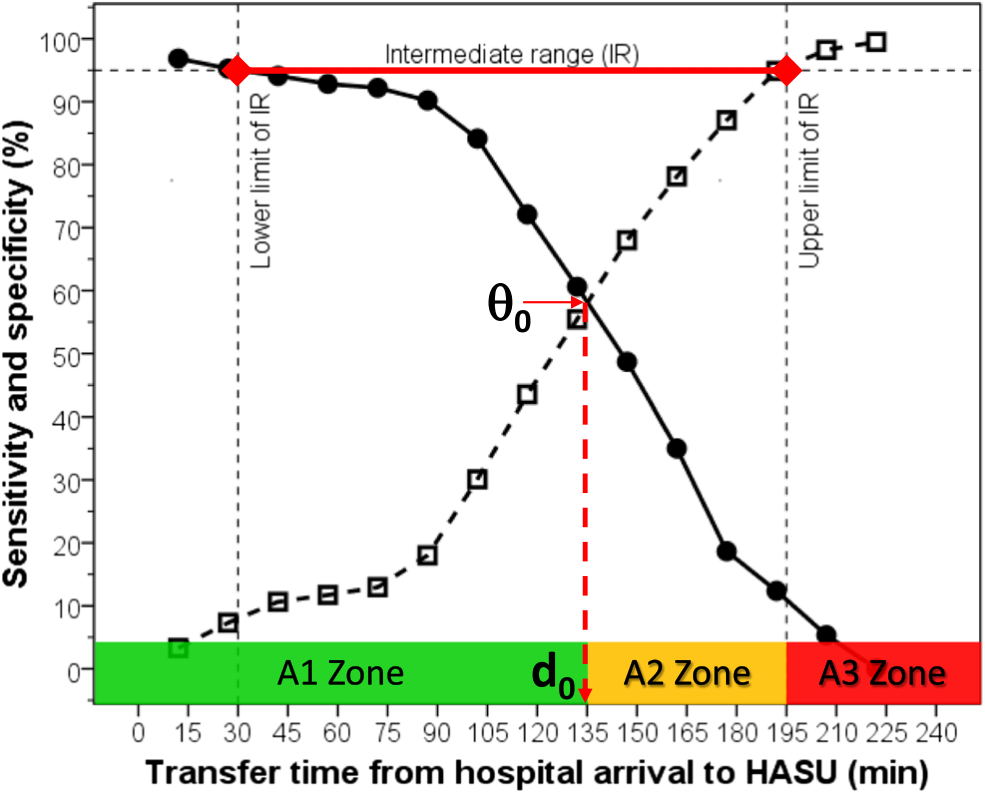
Two-graph ROC plot to identify mortality showing the cut-off of T_Arrival-HASU_ (d_0_) interpolated from the point where sensitivity (●) equals specificity (□) (θ_0_) and from the intermediate range (IR) at 95% level of sensitivity and specificity (red horizontal line)

ROC curve analysis showed that AUC for predicting inpatient mortality was 61% (95%CI: 53–69%, *p =* 0.011) (Fig. 2). The two-graph ROC plot revealed that the d_0_ for identifying inpatient mortality was 2 h/15 min (IR = 30 min to 3 h/15 min) (Fig. 3). Three categories of patients based on these findings were created for further assessment with inpatient mortality by logistic regression; hyper-fast group: 71 (20.2%) patients with T_Arrival-HASU_ within d_0_ (< 2 h/15 min), intermediate group: 143 (40.6%) patients with T_Arrival-HASU_ between d_0_ and upper limit IR (2 h/15 min to 3 h/15 min), and slow group: 138 (39.2%) patients with T_Arrival-HASU_ above upper limit of IR (≥ 3 h/15 min).

Increasing T_Arrival-HASU_ was associated with increasing proportions of inpatient mortality (*χ*^2^ = 7.4, *p* = 0.007): hyper-fast group = 5.6%, intermediate group = 14.0% and slow group = 19.6%. Compared with the hyper-fast group, the slow group had unadjusted OR for inpatient mortality of 4.1 (95%CI:1.4–12.2). This OR increased to 4.9 (95%CI:1.4–17.7) after adjustment for age, sex, co-morbidities, stroke severity, time from symptom onset to arrival, arrival to brain imaging and arrival to thrombolysis. Additional adjustment for time and day on hospital arrival showed the OR increased further to 5.6 (95%CI:1.5–20.6) (Table 2). There was a trend in increasing ORs for those in the intermediate group compared with the hyper-fast group, but was not statistically significant. Extending T_Arrival-HASU_ for the slow group to ≥ 3 h/30 min showed the risk of death elevated to 6.2-fold (95%CI:1.7–23.1) (Table 3).

**Table 2:** Comparison of inpatient mortality between patients with ischaemic stroke who were transferred from arrival to HASU at different time within 4 h (cut-offs at 2 h/15 min and 3 h/15 min)

	Risk of inpatient mortality						
	Hyper-fast group^a^ (< 2 h/15 min)	Intermediate group (≥ 2 h/15 min to 3 h/15 min)			Slow group (≥ 3 h/15 min)		
Mortality rates	4/71 (5.6%)	20/143 (14.0%)			27/138 (19.6%)		
	OR	OR	95% CI	*p*	OR	95% CI	*p*
Model 1: Unadjusted	1	2.72	0.89–8.30	0.078	4.07	1.37–12.15	0.012
Model 2: Adjusted for age, sex, congestive heart failure, hypertension, atrial fibrillation, diabetes and previous stroke	1	2.94	0.93–9.26	0.065	4.24	1.39–13.00	0.011
Model 3: Adjusted as in model 2 plus severity on arrival, time from onset to arrival, arrival to brain imaging and arrival to thrombolysis	1	3.08	0.87–10.91	0.082	4.89	1.35–17.69	0.016
Model 4: Adjusted as in model 3 plus time and day on hospital arrival	1	3.42	0.95–12.32	0.060	5.59	1.52–20.61	0.010

^a^Referent group

**Table 3:** Comparison of inpatient mortality between patients with ischaemic stroke who were transferred from arrival to HASU at different time within 4 h (cut-offs at 2 h/15 min and 3 h/30 min)

	Risk of inpatient mortality						
	Hyper-fast group^a^ (< 2 h/15 min)	Intermediate group (≥ 2 h/15 min to 3 h/30 min)			Slow group (≥ 3 h/30 min)		
	4/71 (5.6%)	25/172 (14.5%)			22/109 (20.2%)		
Model 1: Unadjusted	1	2.85	0.95–8.51	0.061	4.24	1.39–12.88	0.011
Model 2: Adjusted for age, sex, congestive heart failure, hypertension, atrial fibrillation, diabetes and previous stroke	1	3.28	1.00–10.75	0.050	5.46	1.63–18.31	0.006
Model 3: Adjusted as in model 2 plus severity on arrival, time from onset to arrival, arrival to brain imaging and arrival to thrombolysis	1	3.67	1.08–12.46	0.037	6.38	1.78–22.86	0.004
Model 4: Adjusted as in model 3 plus time and day on hospital arrival	1	4.03	1.15–14.19	0.030	6.24	1.68–23.12	0.006

^a^Referent group

We found that compared with the hyper-fast group, the slow group did not have increased risk (adjusted for age, sex, co-morbidities, severity of stroke on arrival and time between procedures) of hospital-acquired pneumonia (OR = 1.88, 95%CI:0.55–6.39, *p* = 0.315) or urinary tract infection (OR = 0.69, 95%CI:0.22–2.19, *p* = 0.529) within 7 days of admission, prolonged length of stay in HASU of > 3 weeks (OR = 1.04, 95%CI:0.45–2.39, *p* = 0.921) or moderately severe to severe disability on discharge (OR = 1.88, 95%CI:0.71–4.96, *p* = 0.203).

Patients presented with moderately severe to severe stroke on arrival were more likely to be thrombolysed earlier (adjusted OR = 0.24, 95%CI:0.10–0.55). Faster thrombolysis on arrival resulted in reduced proportions of patients with moderately severe to severe stroke 24 h post-thrombolysis (i.e. proportions of patients with moderately severe to severe stroke at 24 h post-thrombolysis minus proportions of patients with moderately severe to severe stroke on arrival, *p* = 0.003): − 13.6, − 8.3 and + 4.0% for tertile 1 (< 36 min), tertile 2 (≥ 36 min to 1 h/19 min) and tertile 3 (≥ 1 h/19 min), respectively.

Further analysis was conducted to assess mortality rates in different tertiles of the time between thrombolysis to HASU (i.e. eliminating the influence from the time between arrival and brain imaging and between arrival and thrombolysis). Increasing length of transfer time from thrombolysis to HASU was associated with higher proportions of inpatient mortality (*χ*^2^ = 9.4, *p* = 0.009): tertile 1 (< 1 h/45 min) = 8.2%, tertile 2 (1 h/45 min to 2 h/30 min) = 13.8% and tertile 3 (≥ 2 h/30 min) = 22.4%. Compared with the fastest transfer group, the slowest transfer group had increased risk of inpatient mortality by 3.35-fold (95%CI:1.43–7.89, *p* = 0.006), adjusted for age, co-morbidities and stroke severity on arrival. We found no relationship between time from arrival to brain imaging and inpatient mortality within this group of patients.

## Discussion

The present study focused on the relationship between inpatient mortality and rapidity of transferring patients to HASU from hospital arrival within the recommended 4-h window among patients receiving thrombolysis. Our findings provide evidence-based cut-offs of transfer target below the currently recommended target by NICE and RCP of 4 h, independently of demographic factors, co-morbidities, stroke severity, time to brain imaging, time to thrombolysis and time and day of admission.

We have identified two cut-off levels which provide three alarm time zones to improve stroke survival: “A1 Zone” (hyper-fast T_Arrival-HASU_ < 2 h/15 min) indicates a desirable target for transferring patients to HASU as it has low mortality risk, “A2 Zone” (intermediate T_Arrival-HASU=_2 h/15 min to 3 h/15 min) indicates an increasing risk and should not delay any further, and “A3 Zone” (slow T_Arrival-HASU_ ≥ 3 h:15 min) indicates high mortality risk and should be avoided. This proposal was validated by logistic regression analysis showing that compared with the hyper-fast group in the “A1 Zone”, the slow group in the “A3 Zone” had a 5.6-fold greater adjusted risk of inpatient mortality. We found that among the 138 patients (39.2% of the study sample) in the “A3 Zone”, there were 27 deaths (52.9% of total mortality), compared with only 4 deaths (7.8%) among the 71 patients in the “A1 Zone”. Our findings therefore provide strong support for the use of these time zones.

There exists a number of service standards set by authoritative bodies including NICE [6] and RCP [7] with respect to the acute stroke pathway; these include the time from symptom onset to hospital admission, followed by brain imaging, thrombolysis and transfer to HASU. The recommended 4-h window has been adopted for number of years without modification despite rapid advances in hyperacute stroke service have been achieved in recent years [3]. Brain imaging is now operating continuously 24 h every day with the proportions of patients receiving CT scan within 1 h of hospital arrival rose from 41% in 2013 to 51% in 2016 [8]. Over this 3-year period, “door to needle” time has progressively been quickened by 2 min 20 s a year [8]. Integrated network linking the community and specialist stroke centres has been firmly established [2]. National audits such as SSNAP provide continuous updated stroke service performance and recommendations [8]. The 4-h T_Arrival-HASU_ target therefore may be outdated and a new evidence-based cut-off should now be considered. Further studies are suggested to examine other standards such as time to transfer from the point of thrombolysis to HASU to provide an optimal evidence-based target. The present study did not examine those factors that influence actual T_Arrival-HASU_ times and this is the next step that are practical at both local and regional/national levels. Examples can include, minimising out-of-hours and weekend effects by ensuring that the right capacity remains available to allow seamless flow of patients through the pathway to increase patient throughput and avoid delayed transfer of care.

Additional analysis found that rapid T_Arrival-HASU_ did not significantly associate with a number of adverse outcomes (nosocomial infections within 7 days of admission, prolonged hospital stay or disability on discharge) suggesting that early transfer to HASU not only lowers the rates of mortality but also has no other detrimental effects to the patients. Thus, rapid transfer to HASU provides favourable outcomes to patients treated with thrombolysis for acute stroke. This may in part be explained by a number of benefits gained from faster transfer to HASU including early review from hyperacute stroke specialists and supportive care such as dysphagia screen and appropriate nutrition, which has been shown to associate with better outcomes [9]. We have calculated that dysphagia screen in hyper-fast group was performed within 4 h of admission in 97.2%, between 4 and 72 h in 2.8% and 0% beyond 72 h; in comparison, the corresponding figures for the slow group were 89.9%, 8.0% and 2.2% (*χ*^2^ = 12.6, *p* = 0.007). It should be borne in mind, however, that establishing an ‘optimal’ T_Arrival-HASU_ requires consideration of a number of factors including stability of the patients and trade-offs between benefits and risks in terms of financial costs to accommodate HASU service and safety of the patients when varying the desired true positive rate (sensitivity) and against false positive rate (1-specificity).

Evidence from previous studies has indicated that the earlier is thrombolysis, the greater the likelihood for good stroke outcomes [14, 15]. However, little is known about how stroke severity influences the likelihood of receiving interventions [16]. In this study, we observed that patients with more severe stroke on arrival, indicated by NIHSS score, were more likely to be thrombolysed at earlier times, although they were not necessarily transferred to HASU earlier. Time to stabilise the patient or availability of HASU beds may in part explain this discrepancy. Previous analysis of SSNAP data has found that admission to HASU within 4 h varied with days of the week and there was a variation in mortality with time of the day but this association was not apparent between days of the week in which patients were admitted [17, 18]. Our study found that adjustment for time and day on arrival slightly accentuated the relationship between T_Arrival-HASU_ and mortality, as reflected by an increase in the OR.

### Strengths and limitations

The strengths in the present study lie in their completeness of data including a number of variables, such as time to brain imaging and thrombolysis, which could potentially confound the associations between T_Arrival-HASU_ and mortality. Various cut-offs of T_Arrival-HASU_ could be derived by the powerful two-graph ROC analysis [11, 12] for further examination by logistic regression. Our conclusions are robustly supported by logistic regression analysis where potential confounders were simultaneously adjusted using multivariate modelling as well as further analysis from the point of thrombolysis to HASU. This eliminates the variable periods prior to thrombolysis such time from hospital arrival to brain imaging or from arrival to thrombolysis. We have also extended our analysis to include patients who did not meet the 4-h target of transfer from hospital arrival to HASU and showed that the risk of mortality were similarly elevated among the slowest transfer group. We also explored other cut-offs for T_Arrival-HASU_ including the use of tertiles or limits of IR where sensitivity (lower limit) and specificity (upper limit) were at 90% and found similar, but weaker, patterns in the prediction of inpatient mortality. The present study is limited by its subjects who were restricted to four centres. However, this study population has similar characteristics to those in England and Wales [8] but caution should, however, be taken to extrapolate our findings. The overall mortality rate among patients treated with thrombolysis in the present study (including those who were transferred to HASU beyond 4 h) was 14.8% (64/431) which is lower than the reported figure of 19.4% from a recent meta-analysis of 27 studies [19]. We focused on “T_Arrival-HASU_”, which is as relevant as “door to needle time” or “thrombolysis to HASU” time. It is likely that the outcomes would be similar for these three measures as their journeys are dependent on each other. The reason that we selected T_Arrival-HASU_ is that there is a clear recommended 4-h target transfer window set out by NICE [6] and RCP [7] with which we could compare.

Another limitation of the present study is that, due to the inherent design of SSNAP, other features were not collected that could be associated with clinical outcome in thrombolysed patients, e.g. altered level of consciousness, symptomatic intracranial haemorrhage, presence of large vessel occlusion and site of occlusion. In addition, there is a lack of information about hyperacute complications such as severe hypertension, acute angioedema, hyperacute haemorrhage or subtypes of ischaemic stroke that would be useful for a better understanding of the reasons for increased T_Arrival-HASU_.

In conclusion, T_Arrival-HASU_ below the recommended target window of 4 h associates with lower inpatient mortality. We propose three new alarm time zones to improve stroke survival: “A1 Zone” (T_Arrival-HASU_ < 2 h/15 min) indicates a desirable target for transferring patients to HASU, “A2 Zone” (T_Arrival-HASU=_2 h/15 min to 3 h/15 min) indicates an increasing risk and should not delay any further, and “A3 Zone” (T_Arrival-HASU_ ≥ 3 h/15 min) indicates high risk and should be avoided.
